# Assessment of three-dimensional set-up errors in conventional head and neck radiotherapy using electronic portal imaging device

**DOI:** 10.1186/1748-717X-2-44

**Published:** 2007-12-14

**Authors:** Tejpal Gupta, Supriya Chopra, Avinash Kadam, Jai Prakash Agarwal, P Reena Devi, Sarbani Ghosh-Laskar, Ketayun Ardeshir Dinshaw

**Affiliations:** 1 Department of Radiation Oncology, Advanced Centre for Treatment Research & Education in Cancer (ACTREC), Tata Memorial Centre, Kharghar, Navi Mumbai, India; 2 Department of Radiation Oncology, Tata Memorial Hospital, Parel, Mumbai, India

## Abstract

**Background:**

Set-up errors are an inherent part of radiation treatment process. Coverage of target volume is a direct function of set-up margins, which should be optimized to prevent inadvertent irradiation of adjacent normal tissues. The aim of this study was to evaluate three-dimensional (3D) set-up errors and propose optimum margins for target volume coverage in head and neck radiotherapy.

**Methods:**

The dataset consisted of 93 pairs of orthogonal simulator and corresponding portal images on which 558 point positions were measured to calculate translational displacement in 25 patients undergoing conventional head and neck radiotherapy with antero-lateral wedge pair technique. Mean displacements, population systematic (Σ) and random (σ) errors and 3D vector of displacement was calculated. Set-up margins were calculated using published margin recipes.

**Results:**

The mean displacement in antero-posterior (AP), medio-lateral (ML) and supero-inferior (SI) direction was -0.25 mm (-6.50 to +7.70 mm), -0.48 mm (-5.50 to +7.80 mm) and +0.45 mm (-7.30 to +7.40 mm) respectively. Ninety three percent of the displacements were within 5 mm in all three cardinal directions. Population systematic (Σ) and random errors (σ) were 0.96, 0.98 and 1.20 mm and 1.94, 1.97 and 2.48 mm in AP, ML and SI direction respectively. The mean 3D vector of displacement was 3.84 cm. Using van Herk's formula, the clinical target volume to planning target volume margins were 3.76, 3.83 and 4.74 mm in AP, ML and SI direction respectively.

**Conclusion:**

The present study report compares well with published set-up error data relevant to head and neck radiotherapy practice. The set-up margins were <5 mm in all directions. Caution is warranted against adopting generic margin recipes as different margin generating recipes lead to a different probability of target volume coverage.

## Background

Set-up errors, though undesirable are an inherent part of the radiation treatment process. They are defined as the difference between the actual and intended position with respect to radiation delivery. Coverage of target volume is a direct function of set-up margins, which should be optimized to prevent inadvertent irradiation of adjacent normal tissues. Planning target volume (PTV) that encompasses the clinical target volume (CTV) with some margins to account for such uncertainties in patient positioning, organ motion, and beam geometry is universally accepted today as the benchmark for radiotherapy (RT) dose prescription [1,2]. The use of portal imaging to measure set-up errors is accepted standard practice [3]. The widespread availability of electronic portal imaging devices (EPID), coupled with a demand to reduce PTV margins, particularly for high-precision radiotherapy has provided impetus for such assessments across the radiation oncology community [4]. The experience, training, commitment and time available with radiation therapy staff can have a major impact on daily positioning accuracy. It is generally recommended that every institution generate data on its set-up accuracy without blindly adopting published margin recipes. It is in this context that this study was planned at a newly commissioned academic radiotherapy unit of a comprehensive cancer center.

### Aims and objectives

The primary objective of this study was to assess the set-up accuracy of head and neck RT using customized thermoplastic immobilization and compare with 'state-of-the-art' practices. A secondary objective was to define an optimal three-dimensional (3D) CTV-PTV margin prior to the clinical implementation of high-precision conformal techniques for head and neck radiation therapy.

## Methods

Patients receiving post-operative adjuvant RT for a head and neck cancer on a Linear Accelerator (LA) equipped with a camera-based EPID were considered for inclusion in the study. Only patients receiving RT with antero-lateral portals were included. Patients treated with bilateral fields were excluded, as their anterior reference image was not available. Only patients with at least 3 sets of orthogonal portal images were included in the dataset. A total of 25 patients met the inclusion criteria on which 186 images and 558-point positions were available for analysis. Rotational errors were not assessed in this study.

### Immobilization and simulation

For the purpose of simulation and subsequent treatment, patients were immobilized in supine position on a four clamp base plate with customized thermoplastic mask on an appropriate neck rest. Radiation fields were simulated and optical field projection was marked on the thermoplastic mould for subsequent positioning and treatment. The anterior and lateral simulator images were transferred to LANTIS^® ^(version 6.1, Siemens Medical Solutions, Concord, CA, USA). These served as reference images for comparison with the portal images.

#### Portal imaging and evaluation

Portal images were acquired using BEAMVIEW^® ^(version 2.2, Siemens Medical Solutions, Concord, CA, USA). This is a camera-based EPID system consisting of a detector screen, its light enclosure, optical chain, camera and video capture [3]. It is mounted iso-centrically on the LA with a detector size of 35 × 44 cm. EPID images were acquired at a reduced dose rate of 100 Monitor Units (MU) per minute and 4–8 MUs were delivered per field for portal acquisition. A double exposure portal image of the anterior and lateral fields was obtained. For each patient 3–6 (median 4) portal images per field were acquired during the course of fractionated RT. The small dose delivered by portal imaging was not taken into consideration in calculating the final total dose received by any patient. Reference images from Simulix HQ^® ^(Nucletron BV, Veenendaal, Netherlands) were used for comparison with the portal images. As BEAMVIEW^® ^does not have image automatic overlaying and fusion ability, evaluation of translational set-up errors was done by defining two reproducible and easily identifiable bony landmarks in upper and lower part of the treatment field each in anterior and lateral images. After demonstration of the technique by a radiation oncologist, one radiation therapy technologist carried out all the measurements to avoid inter-observer variation. A radiation oncologist randomly checked 5% of all displacements and re-verified measurements in case of outliers during the process of image analysis. Five sets of orthogonal portal images were randomly selected for manual overlay and verification on a graph paper after appropriate scaling. There was reasonable agreement between the digital and manual measurements suggesting reliability of the technique. For the purpose of documentation and analysis anterior, superior, and right-sided shifts were coded as positive shifts and posterior, inferior, and left-sided shifts as negative shifts. Some of the potential sources of errors such as laser alignment, display accuracy, iso-centric accuracy and jaw reproducibility were not taken into consideration for the final match result. It was assumed that the routine periodic quality assurance employed for the LA would ensure minimal impact of the aforesaid on daily set-up. Statistical Package for Social Sciences (SPSS version 14.0) and Microsoft Office Excel (MS Office 2003) were used statistical analysis.

## Results and observations

### Translational displacement

**Figure 1:**
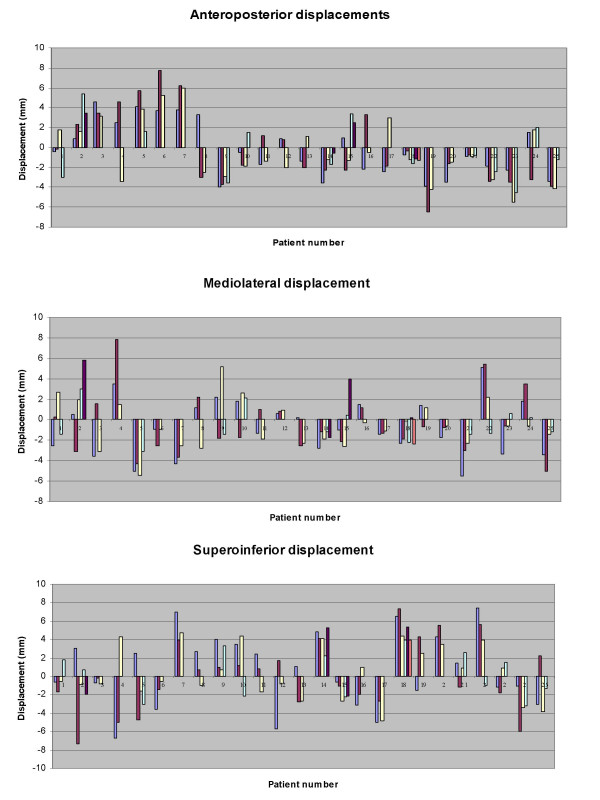
Patient-wise distribution of set-up deviation in all three directions.

**Figure 2:**
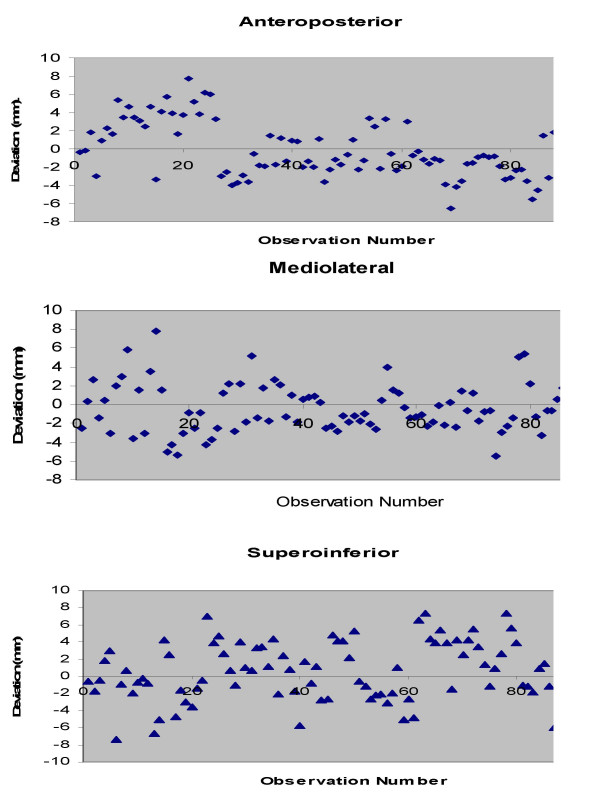
Scatterplot of translational displacements for all observations in all three directions.

**Figure 3:**
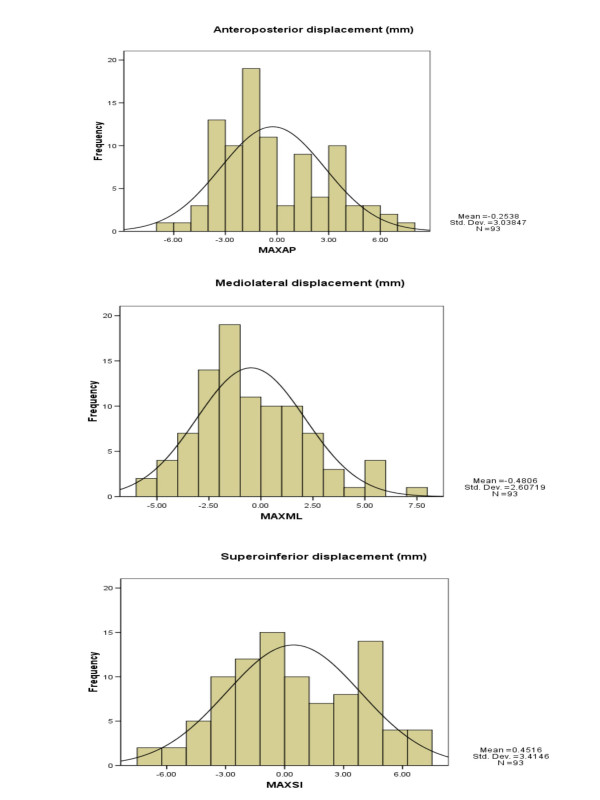
Histogram of translational displacements in all three directions including mean and standard deviation.

Translational displacements were measured in 186 (93 anterior and 93 lateral) portal images and assessed over 558-point positions in antero-posterior (AP), medio-lateral (ML) and supero-inferior (SI) direction. The mean displacement in AP; ML; and SI direction was -0.25 mm (range -6.50 to +7.70 mm); -0.48 mm (range -5.50 to +7.80 mm); and +0.45 mm (range -7.30 to +7.40 mm) respectively (Fig. 1,2 and 3). The set-up errors in AP and ML direction were normally distributed (skewness ≤ 2 × standard error of skewness), whereas they were skewed inferiorly in the SI direction. Ninety three percent of the set-up deviations were within 5 mm in all three directions.

### Systematic and random errors

Systematic (Σ) and random (σ) errors were calculated as per conventionally defined norms [5,6]. The systematic component of the displacement represents displacement that was present during the entire course of treatment. For an individual patient, the systematic displacement was assessed by mean values of all the displacements and for the whole population the systematic error was represented by the standard deviation (SD) from the values of mean displacement for all individual patients. The random errors represent day-to-day variation in the set-up of the patient. For each patient, dispersion around the systematic displacement was calculated to assess the random displacement. For the whole population, the distribution of random displacements was expressed by the root mean square of SD of all patients. The population systematic error (Σ) in AP; ML; and SI direction was 0.96, 0.98 and 1.2 mm respectively. The population random error (σ) in the corresponding directions was 1.94, 1.97 and 2.48 mm respectively. 3D vector length was calculated for every patient and averaged to give the mean 3D vector of displacement. The mean 3D vector of displacement was 3.84 mm.

### Margin calculation

CTV-PTV margins were calculated using the International Commission on Radiation Units and Measurements (ICRU) Report 62 [2], Stroom's [6,7], and van Herk's [8,9] formulae (Table 1). Using the ICRU recommendation, the CTV-PTV margin in the AP; ML; and SI direction was 2.16, 2.20, and 2.76 mm respectively. The corresponding values were 3.28, 3.34 and 4.14 mm with Stroom's formula and 3.76, 3.83 and 4.74 mm with van Herk's formula (Table 1).

**Table 1:** Population systematic and random errors and necessary CTV to PTV margins

**Population set-up errors**			**CTV to PTV margins (mm)**		
***Direction***	***Systematic (Σ)***	***Random (σ)***	***ICRU 62 (Sqrt Σ*^2 ^+ *σ*^2^)**	***Stroom (2 Σ *+ *0.7σ)***	***van Herk (2.5 Σ *+ *0.7σ)***

Antero-Posterior (AP)	0.96	1.94	2.16	3.28	3.76
Medio-Lateral (ML)	0.98	1.97	2.20	3.34	3.83
Supero-Inferior (SI)	1.20	2.48	2.76	4.14	4.74

## Discussion

This report attempts to evaluate the set-up accuracy in patients receiving conventional radiotherapy for head and neck cancers with antero-lateral portals at a newly commissioned academic radiotherapy unit of a comprehensive cancer centre using a camera-based portal imaging system. Unlike other commercially available software, BEAMVIEW^® ^is not equipped with anatomy matching and image fusion module. Hence, image analysis was carried out by comparing the reference simulator image with portal image using fixed bony landmarks, a good surrogate for target localization in head and neck cancers [4]. As there exists a possibility of variation in manual measurements two different points were used for evaluation of displacements in each direction. Furthermore, comparing online digital measurements with manual measurements using printouts of portal images validated the technique. Emphasis was laid on the technique of manual measurements by precisely choosing the same points on reference and portal images. Random cross checking by a radiation oncologist ensured the quality of image analysis. The set-up errors in AP and ML direction were normally distributed (skewness ≤ 2 × standard error of skewness), whereas they were skewed inferiorly in the SI direction. Ninety three percent of the set-up deviations were within 5 mm in all three directions. The CTV to PTV margins were within 5 mm in all directions. This compares reasonably well with the published head and neck data using head cast and thermoplastic immobilization devices. Population systematic (Σ) and random errors (σ) also correlated well with the published literature (Table 2) [10-16]. However, they were larger than those achieved by Humphrey et al [14] using Cabulite customized shell.

**Table 2:** Population systematic (Σ) and random (σ) errors of selected contemporary series and correlation with probability of target volume coverage

**Series**	**Σ**	**σ**	**Displacements or errors**
*Hess [10]*	Not reported	Not reported	3 mm for 50% coverage
			9 mm for 95% coverage
*Bentel [11]*	Not reported	Not reported	5–10 mm (87–90% with 5 mm margin)
*Gibeau [12]*	1 – 2.2	0.7 – 2.3	4.5–5.5 mm for 90%probability of target coverage
*De Boer [13]*	1.5 – 2.0	1.5 – 2.0	Probability values not specified
*Humphrey [14]*	0.02 – 0.9	0.4 – 0.7	3 mm for 95% of the errors.
			5 mm for 99% of errors
*Zhang [15]*	1.5 – 3.2	1.1 – 2.9	5.5 mm for 90% probability of target coverage
*Suzuki [16]*	0.7 – 1.3	0.7 – 1.6	5 mm margin for PTV and 3 mm for PRV
			Probability values not specified
*Present Study*	0.96 – 1.2	1.94 – 2.48	93% displacements within 5 mm
			<5 mm CTV-PTV margin in all directions

Several mathematical formulae have been recommended for generating CTV-PTV margins. Coverage of target volume is a direct function of the set-up margin, which should be optimized to prevent inadvertent irradiation of adjacent normal tissues that may precipitate unwarranted radiation morbidity. The ICRU 62 [2] states that systematic and random uncertainties should in an ideal approach be added in a quadrature, which should then be used for margin calculation. However, this approach assumes that random and systematic errors have an equal effect on dose distribution, which may not necessarily be the case. Random errors blur the dose distribution whereas systematic errors cause a shift of the cumulative dose distribution relative to the target. In fact, it has been consistently shown that systematic errors are of higher dosimetric consequences than random errors. Using coverage probability matrices and dose-population histograms, Stroom et al [6] and Van Herk et al [9] have suggested formulae incorporating this differential effect. Stroom's margin recipe (2Σ + 0.7σ) ensures that on an average, 99% of the CTV receives more than or equal to 95% of the prescribed dose. The formula by van Herk (2.5Σ + 0.7σ) seems to be the most appropriate as it ensures that 90% of patients in the population receive a minimum cumulative CTV dose of at least 95% of the prescribed dose. The CTV to PTV margins using van Herk's formula were 3.76, 3.84, and 4.74 mm in AP; ML; and SI direction respectively.

As stated, some of the published margin-generating recipes do not differentiate between random and systematic errors. Caution should be exercised while comparing data from different series as each group has used different model parameters to derive cumulative set-up errors. Different margin generating recipes lead to a different probability of target volume coverage in different population setting depending on the distribution of shifts. It is therefore suggested that before adopting any published margin recipe, factors that can potentially impact upon margins should also be taken into consideration.

A major drawback of the study was the lack of automatic anatomy matching and image fusion facilities in BEAMVIEW^®^, which could have resulted in reduction in the accuracy of measurements. However, an attempt was made to compensate for this by manually verifying measurements using appropriately scaled printouts on graph paper. Secondly, this study did not attempt to measure rotational errors or intra-fraction displacements.

The good set-up accuracy comparable with published literature [5] achieved hereof for conventional head and neck radiotherapy is also a reflection of the experience, training, commitment, and time available with radiation therapy staff at an academic radiotherapy unit that treats patients only on approved clinical trials. The 3D mean displacements though comparable with previously published literature, had a wide range at times leading to high individual displacements (>7 mm also). This would be unacceptable for high-precision techniques. Attempts are being made to reduce such errors by incorporating offline correction strategies whenever displacements are >3 mm in any direction. Furthermore, a commercially available infrared positioning system is also being prospectively evaluated to increase the set-up accuracy particularly for high-precision conformal techniques. An alternative method of improving the repositioning accuracy would be the use of indexed patient positioning systems and fixed couch inserts.

Image-guided radiation therapy (IGRT) is an innovative and exciting approach for set-up verification that can be potentially useful for high-precision techniques with inherently conformal dose distributions and sharp dose gradients. Contemporary IGRT systems allow accurate internal target positioning and even real-time tumour tracking with a potential to substantially reduce margins. In-room image-guidance systems are either gantry mounted or floor/ceiling mounted. The strategies for IGRT include the use of a) orthogonal radiographs either alone or in conjunction with infrared marker tracking, b) ultrasound imaging with or without implanted fiducial markers, and c) kilovoltage or megavoltage fan-beam or cone-beam computed tomography for volumetric imaging. The reader is referred to an excellent contemporary review on this topic [17].

## Conclusion

The present study is a report on the set-up accuracy of patients receiving conventional head and neck radiotherapy that compares well with published set-up error data. Ninety three percent of translational displacements were within 5 mm. The set-up margins were <5 mm in all three directions. It is suggested that before adopting any published margin recipe, factors that can potentially impact upon margins should also be taken into consideration to ensure adequacy of target volume coverage.

## Competing interests

The author(s) declare that they have no competing interests.

## Authors' contributions

TG conceived the study, did data analysis & interpretation, and wrote final manuscript. SC was involved in data collection & analysis, literature search, and manuscript preparation. AK executed the study and helped in data collection. JPA did the literature search and helped in manuscript preparation. RDP was involved in study execution and data collection. SGL and KAD did a critical review of manuscript. All authors read and approved final manuscript.
